# Rough-type and loss of the LPS due to *lpx* genes deletions are associated with colistin resistance in multidrug-resistant clinical *Escherichia coli* isolates not harbouring *mcr* genes

**DOI:** 10.1371/journal.pone.0233518

**Published:** 2020-05-20

**Authors:** Mojtaba Moosavian, Nasrin Emam, Daniel Pletzer, Mohammad Savari

**Affiliations:** 1 Department of Microbiology, School of Medicine, Ahvaz Jundishapur University of Medical Sciences, Ahvaz, Iran; 2 Infectious and Tropical Diseases Research Center, Health Research Institute, Ahvaz Jundishapur University of Medical Sciences, Ahvaz, Iran; 3 Department of Microbiology and Immunology, School of Biomedical Sciences, University of Otago, Dunedin, New Zealand; Nitte University, INDIA

## Abstract

The emergence of multidrug-resistant *Escherichia coli* has become a great challenge in treating nosocomial infections. The polymyxin antibiotic colistin is used as a ‘last-line’ therapy for such strains, but resistance to colistin is increasingly emerging all over the world. In this study, we investigated lipopolysaccharides (LPS) of colistin-resistant isolates and examined mutations in *lpx* genes in strains not harbouring *mcr* genes. We examined 351 clinical *E*. *coli* isolates with 38 showing reduced susceptibility to colistin. These isolates were collected from different clinical specimens including blood, urine, and wounds, but no stool. After confirmation of the isolates via a BD Phoenix-100 system (Becton Dickinson, USA), we performed antimicrobial susceptibility tests to characterize the resistance pattern of these isolates to different classes of antibiotics, using the disk diffusion test. The Minimum Inhibitory Concentration (MIC) of colistin was determined using E-test strips. The presence of mobile colistin resistance (*mcr-1* and *mcr-2*) genes was tested for all isolates. LPS (including lipid A) were extracted from all isolates and associated *lpx* genes analyzed by PCR and sequencing. Among the 38 clinical *E*. *coli* isolates with reduced susceptibility to colistin, 52% were resistant to colistin. The MICs of colistin ranged from 0.5 μg/ml to ˃256 μg/ml. Within the 20 colistin-resistant strains, six isolates carried the *mcr-1* gene, but not *mcr-2*. Heterologous expression of the *mcr-1* gene in susceptible *E*. *coli* DH5α increased the MIC of colistin by eight-fold. The remaining 14 isolates, were negative for both *mcr* genes. Six isolates were further negative for LPS production and five showed rough LPS phenotypes. Here we present evidence that loss of LPS or lipid A-deficiency can lead to colistin-resistance in clinical *E*. *coli* isolates not harbouring *mcr* genes.

## Introduction

Multidrug-resistant (MDR) *Enterobacteriaceae* such as *Escherichia coli* are associated with life-threating infections, particularly in hospitalized and immunocompromised patients. Those suffering from urinary tract infections, bacteremia, or lung infections are especially at risk [1, 2].

The first treatment choice of infections caused by MDR Gram-negative bacteria are carbapenem antibiotics (i.e., antibiotics inhibiting cell wall synthesis) [3–5]. However, resistance to this class of antimicrobials is steadily increasing [6]. The antibiotic Colistin (polymyxin E) is a 60-years old antibiotic that is considered a "last resort" antibiotic used for infections caused by MDR Gram-negative bacteria including the Carbapenem resistant strains [7]. Polymyxin antibiotics interact with the lipopolysaccharides (LPS) of the outer membrane of Gram-negative bacteria to displace the divalent cations; especially Ca^2+^ and Mg^2+^ through a competitive process [8]. LPS is typically composed of three distinct regions: the lipid A (toxic component), the core oligosaccharide, and the O-antigen polysaccharide (serotype component), forming so-called smooth LPS (sLPS). In contrast, rough LPS (rLPS) producing strains lack or have reduced O-antigens [9, 10].

Lipid A is the main target of polymyxin antibiotics. Lipid A biosynthesis in *E*. *coli* involves several different genes: *lpxA* (encoding a UDP-N-acetylglucosamine acyltransferase) catalyzes the first reaction of lipid A biosynthesis, *lpxB* (encoding a lipid-A-disaccharide synthase) catalyzes the fifth step in lipid A biosynthesis, *lpxC* (encoding a UDP-3-O-[3-hydroxymyristoyl] N-acetylglucosamine deacetylase) catalyzes the second reaction and the first committed step in lipid A biosynthesis and *lpxD* (encoding a UDP-3-O-[3-hydroxymyristoyl] glucosamine N-acyltransferase) catalyzes the third step of lipid A biosynthesis [11]. Furthermore, *lpxH* (encoding a UDP-2,3-diacylglucosamine hydrolase) catalyzes the fourth step in lipid A synthesis [12], *lpxK* (encoding a Tetraacyldisaccharide 4’-kinase) catalyzes the sixth step in lipid A biosynthesis [13], *lpxL* (encoding a lauroyl acyltransferase) incorporates a laurate residue into KDO2-lipid IVA from lauroyl-ACP [14] and *lpxM* (encoding a myristoyl-acyl carrier protein-dependent acyltransferase) [15].

The general mechanisms behind colistin resistance include LPS modifications, LPS removal [16], and uptake of plasmids carrying *mcr* genes [17]. In many Gram-negative bacteria, modifications of lipid A by addition of 4-amino-4-deoxy-L-arabinose (L-Ara4N) and/or phosphoethanolamine (PEtn) act to reduce the net LPS negative charge, thereby increasing resistance to colistin [18]. The complete loss of LPS due to *lpx* gene(s) interruption or deletion, also causes colistin resistance [18]. In addition, strains acquiring plasmids that carry *mcr* genes (i.e., mobilized colistin resistance genes), encoding phosphatidylethanolamine transferase that can modify the LPS by adding a phosphatidylethanolamine residue to lipid A component, have been emerging globally [19]. To date, nine mobilized colistin resistance genes (*mcr-1* to *mcr-9*) have been identified. They are homologous to each other and work in similar ways and among these, *mcr-1* and *mcr-2* are the most prevalent genes [20–22].

*Enterobacteriaceae* strains with single mutation in genes associated with colistin resistance remain susceptible to the Colistin. However, the MICs of the Colistin for these strains are markedly increased (i.e., they have reduced susceptibility) [23].

Previously, we reported on the emergence of clinical, colistin-resistant *E*. *coli* isolates which some were harbouring and some not harbouring *mcr-1* genes [24]. Here, we further investigated the LPS structure of colistin-resistant isolates and examined mutations in *lpx* genes in strains not harboring *mcr* genes.

## Materials and methods

### Bacterial isolates

A total of 351 isolates of *E*. *coli* were detected in infectious-associated *Enterobacteriaceae* spp. [23] isolated from different clinical specimens such as blood, urine, or wounds. These isolates were collected from the laboratories of the teaching hospitals (Golestan, Aboozar and Razi) related to Ahvaz Jundishapur University of Medical Sciences, Ahvaz, Iran from April to June 2017. This study was approved by the Institutional Review Board (IRB) and Ethics Committee of the Ahvaz Jundishapur University of Medical Sciences. Bacterial isolates were collected as a part of routine patient care, and all patient data were anonymized before the researchers gained access. Species identification was performed using a BD Phoenix-100 system (Becton Dickinson, USA) and confirmed by 16S rRNA gene sequencing.

### Drug Susceptibility Testing (DST)

DST was performed using the disk diffusion method (Kirby-Bauer) on Mueller-Hinton agar (Merck, Germany) plates according to the Clinical and Laboratory Standards Institute (CLSI) guidelines [25]. The tested antibiotic disks were: colistin (COL, 10μg), ciprofloxacin (CIP, 5μg), tetracycline (T, 30μg), imipenem (IMI, 10μg), ceftazidime (CAZ, 30μg), azithromycin (AT, 15μg), and amikacin (AK, 30μg) (MAST Co., UK). The phenotype of *E*.*coli* was defined as MDR according to the International Expert proposal for Interim Standards Guidelines [26]. Minimum Inhibitory Concentration (MIC) of colistin (Sigma-Aldrich, USA) was determined using the E-test strips (Mast, UK) method according to CLSI guidelines.

### DNA extraction

The DNA was extracted using the High Pure PCR Template Preparation Kit (Roche Inc., Mannheim, Germany) following the manufacturer’s instruction.

### Detection of *mcr-1* gene

*mcr-1* was amplified using the primers, *mcr-1*F: CGGTCAGTCCGTTTGTTC and *mcr-1*R: CTTGGTCGGTCTGTAGGG [24], and the following conditions: The master mix was prepared in a final volume of 25 μL containing 10× PCR buffer, 50 mM MgCl_2_, 10 mM dNTPs, 10 μM of each primer, 5 U/μL of Taq DNA polymerase and 5 μL of extracted DNA as a template. The DNA amplification was performed based on the following program: initial denaturation at 94°C for 5 mins, 25 cycles of denaturation at 94°C for 1 min, annealing at 51°C for 30 s, extension at 72°C for 30 s and a final extension at 72°C for 5 mins. DNA from *E*. *coli* KP81 and KP37 strains harbouring *mcr-1* and *mcr-2*, respectively, were used as the positive controls. Genomic DNA from colistin-susceptible *E*. *coli* ATCC 25922 was used as a negative control. The PCR products were separated by electrophoresis on a 1.5% agarose gel containing 0.5 μg/ml ethidium bromide. The bands were visualized under UV light using a gel documentation system (Protein Simple, Santa Clara, CA, USA). All PCR products were sequenced and compared the strain information from GenBank. The partial sequence of *mcr-1* from *E*. *coli* strain ECajums9 have been deposited to the GenBank under the accession number of MH627973.

### Cloning

To evaluate the role of *mcr-1* in colistin resistance, we heterologously expressed this gene in a colistin-susceptible *E*. *coli* strain. For this purpose, the coding sequence of the *mcr-1* gene (MF084991.1 (was synthesized and cloned under the *lac* promoter into pBluescript (GenScript). The pBluescript.*mcr-1* recombinant plasmid was transformed into *E*. *coli* DH5α and clones selected on LB agar plates containing 50 μg/ml ampicillin and 2 μg/ml colistin. Functional expression of the cloned *mcr-1* gene in *E*. *coli* DH5α was further verified via MIC experiments. *E*. *coli* DH5α carrying the pBluescript vector without the *mcr-1* genes was used as a control.

### Extraction of LPS

The LPS from colistin-resistant isolates and a colistin susceptible standard strain (*E*. *coli* strain ATCC 25922) was extracted using the LPS Extraction Kit (iNtRON Biotechnology, South Korea) according to the manufacturer’s instructions. The purity of extracted LPS was evaluated by silver staining of SDS-PAGE gels and HPLC analysis. Limulus Amebocyte Lysate (LAL) and rabbit pyrogen tests were done to evaluate the functionality of the purified LPS.

### SDS-PAGE and silver nitrate staining

The purified LPS was solubilized in a sample buffer to the desired concentration (1 mg/mL). 10 μL/well from each sample was separated on a 12% resolving gel with a 4% stacking gel under reducing condition at 100 *mA* for 2 h using mini-PROTEAN electrophoresis instrument (Bio-Rad Laboratories, California, USA). Silver staining of the gels was performed according to the standard protocol [13].

### *lpx* genes operon analysis

*lpx* genes were amplified using a Thermocycler (Eppendorf, Germany) and the primers listed in Table 1.

**Table 1:** Sequences of primers used for detection of *lpxA*, *lpxB*, *lpxC and lpxD* genes.

Target gene	Primer sequence (5’ 3’)	Size (bp)	Annealing Temperature (°C)	Ref.
*lpxA-*F	ACCATTCATCGTGGCACAGT	356	54	This study
*lpxA-*R	ATAGCGGTAATCGCCTCACG			
*lpxB-*F	AAAGAACATGTGCCCAACGC	458	54	This study
*lpxB-*R	CGGGCGGCATTTTTATCTGG			
*lpxC-*F	TACCGGCAAGAAAGTCACCC	689	54	This study
*lpxC-*R	TGAACAAGTCACCGATCGCA			
*lpxD*-F	TGATTGAGTCCGGCGTTGAA	574	54	This study
*lpxD*-R	CGCCAGACTTTGTTGGGTTG			

The Master mix was prepared in a final volume of 25 μl containing 10X PCR Buffer, 50 mM MgCl_2_, 10 mM dNTPs, 10 μM of each primer, 5 U/μl of Taq DNA Polymerase, 10 ng of the genomic DNA was used as the template. The amplification was performed with the following program: initial denaturation at 94°C for 5 min, 25 cycles of denaturation at 94°C for 1 minute, annealing at 54°C for 30 seconds, extension at 72°C for 30 seconds and a final extension at 72°C for 5 minutes. Genomic DNA from *E*. *coli* ATCC 25922 was used as control. The PCR products were separated by electrophoresis on a 1.5% agarose gel containing 0.5 μg/ml Sybr Safe DNA Gel staizn (Thermo Fisher, USA). The bands were visualized under UV light using a gel documentation system (Protein Simple, USA). The partial sequence of *lpxA*, *lpxB*, *lpxC*,and *lpxD* from *E*. *coli* strain ECajums1, ECajums9, ECajums26 and ECajums30 have been deposited to the GenBank under the accession number of. Multiple Sequence Alignment (MSA) was done using the MEGA-X software.

### Construction of genetic maps

Two genetic maps; the *lpx* genes cluster (*lpxA*, *lpxB*, *lpxC*, *lpxD*, *lpxM*, *lpxL*, *lpxK*, and *lpxH*) and the adjacent genes between *lpxA*, *lpxB*, *lpxC*, *lpxD* genes were constructed using the SnapGene software (version 3.2.1) and the data from Genbank (NZ_CP032667.1) and shown in Figs 3 and 4.

### Statistical analysis

Statistical analysis was performed using the “IBM SPSS statistics 22” software (IBM analytics; USA). Statistical significance of variables determined by chi-square and Fisher’s exact tests and statistical significance determined with a p-value ≤ 0.05.

## Results and discussion

We previously identified 351 clinical *E*. *coli* strains where 10.8% (n = 38) showed reduced susceptibility to colistin based on a disk diffusion assay. Antibiotics resistance profiles showed that 84.2% of the isolates (n = 32) were MDR (Table 2).

**Table 2:** The characteristics of colistin resistant *E*. *coli* clinical isolates tested in this study.

*E*. *coli* isolates	*Colistin MIC (*μg/m*l)*	*LPS type*	*lpxA* gene	*lpxB* gene	*lpxC* gene	*lpxD* gene	*mcr-1*	Resistance pattern (Kirby-Bauer)
ECajums1	1.5	S	+	+	+	+	-	T, CIP,CAZ
ECajums2	1.5	S	+	+	+	+	-	AT
ECajums3	6	S	+	+	+	+	-	CAZ
ECajums4	0.5	S	+	+	+	+	-	-
ECajums5	0.75	S	+	+	+	+	-	AK, CIP, T, CAZ, AT
ECajums6	0.25	S	+	+	+	+	-	AK, CIP, T, CAZ, AT
ECajums7	0.75	S	+	+	+	+	-	T, AT, CAZ
ECajums8	0.75	S	+	+	+	+	-	CAZ
ECajums9	2.5	S	+	+	+	+	+	T, AT, CAZ
ECajums10	4	S	+	+	+	+	+	T, CAZ
ECajums11	˃256	R	-	+	-	-	-	AK, CIP, T, CAZ, IMI, AT
ECajums12	˃256	ND	-	-	-	-	-	AK, CIP, T, CAZ, AT
ECajums13	1.5	ND	-	-	-	-	-	AT, CIP, CAZ
ECajums14	1	S	+	+	+	+	-	T, AK, CIP, CAZ
ECajums15	8	ND	-	-	-	-	-	AT, T, CAZ, IMI
ECajums16	˃256	ND	-	-	-	-	+	AT, T, CAZ, IMI
ECajums17	˃256	R	-	-	-	-	-	CAZ, IMI
ECajums18	˃256	R	-	-	-	-	-	T, AT, CAZ, IMI
ECajums19	1.5	ND	-	-	-	-	-	T, AT, CAZ, IMI
ECajums20	1.5	S	+	+	+	+	-	T, CIP, IMI, CAZ
ECajums21	32	S	+	+	+	+	+	CAZ, IMI
ECajums22	3	ND	-	-	-	-	-	AK, T, CAZ, IMI
ECajums23	˃256	R	-	+	+	+	-	CAZ, IMI
ECajums24	1	S	+	+	+	+	-	T, AT, CIP, CAZ, IMI
ECajums25	2	R	-	-	-	-	+	T, CIP, CAZ, IMI
ECajums26	˃256	S	+	+	+	+	+	AT, T, CAZ, IMI, CIP
ECajums27	1.5	S	+	+	+	+	-	CAZ
ECajums28	1	S	+	+	+	+	-	T, CIP, CAZ
ECajums29	2.5	S	+	+	+	+	-	T, CIP, CAZ
ECajums30	4	S	+	+	+	+	-	AT, IMI, CIP
ECajums31	2.5	S	+	+	+	+	-	T, CIP, IMI, CAZ
ECajums32	6	S	+	+	+	+	-	T, CAZ
ECajums33	0.75	S	+	+	+	+	-	AT, T, CAZ
ECajums34	1	S	+	+	+	+	-	AT, CIP
ECajums35	1.5	S	+	+	+	+	-	AT
ECajums36	0.5	S	+	+	+	+	-	T, CIP, CAZ
ECajums37	12	S	+	+	+	+	-	AK, CIP, T, CAZ, IMI, AT
ECajums38	2	S	+	+	+	+	-	AT, CIP

Abbreviations: S: smooth-LPS, R: rough-LPS, ND: none detected, CAZ: ceftazidime, T: tetracycline, AT: azithromycin, CIP: ciprofloxacin, IMI: imipenem, AK: amikacin.

**Fig 1:**
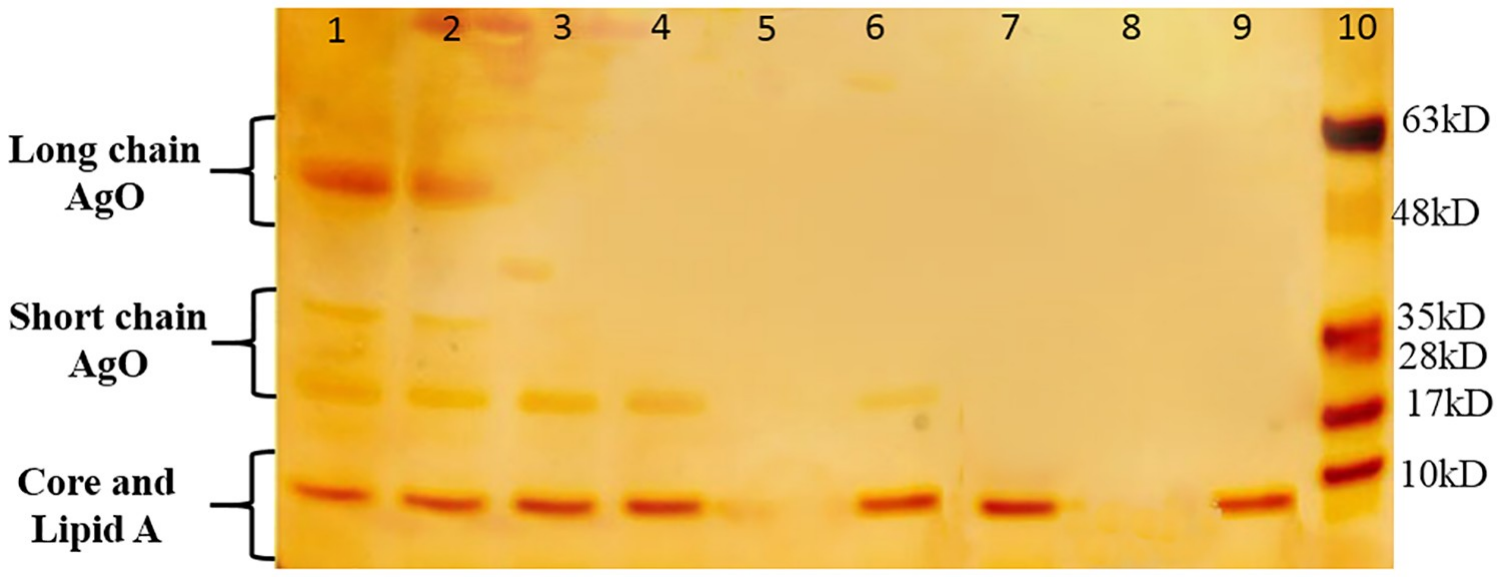
LPS structure analysis of *E*. *coli* wild type and mutants. SDS-PAGE analysis of the LPS fractions from Lane 1: positive control with sLPS (*E*. *coli* type strain ATCC 25922); Lane 2: colistin-susceptible clinical isolate (ECajums5, which shows sLPS); Lane 3, 4, 6, 7, 9: rLPS (ECajums11, ECajums17, ECajums18 and ECajums23); Lane 5, 8: colistin-resistant clinical isolates without LPS (ECajums12 and ECajums15); Lane 10: molecular mass markers. sLPS: smooth LPS, rLPS: rough LPS.

Here, we further studied colistin resistance of these 38 isolates and found that only 52.6% (n = 20) were indeed colistin-resistant according to the CLSI breakpoint (MIC ≥ 2 μg/ml). The 18 colistin-susceptible isolates were further investigated regarding the presence of smooth or rough LPS (Fig 1).

**Fig 2:**
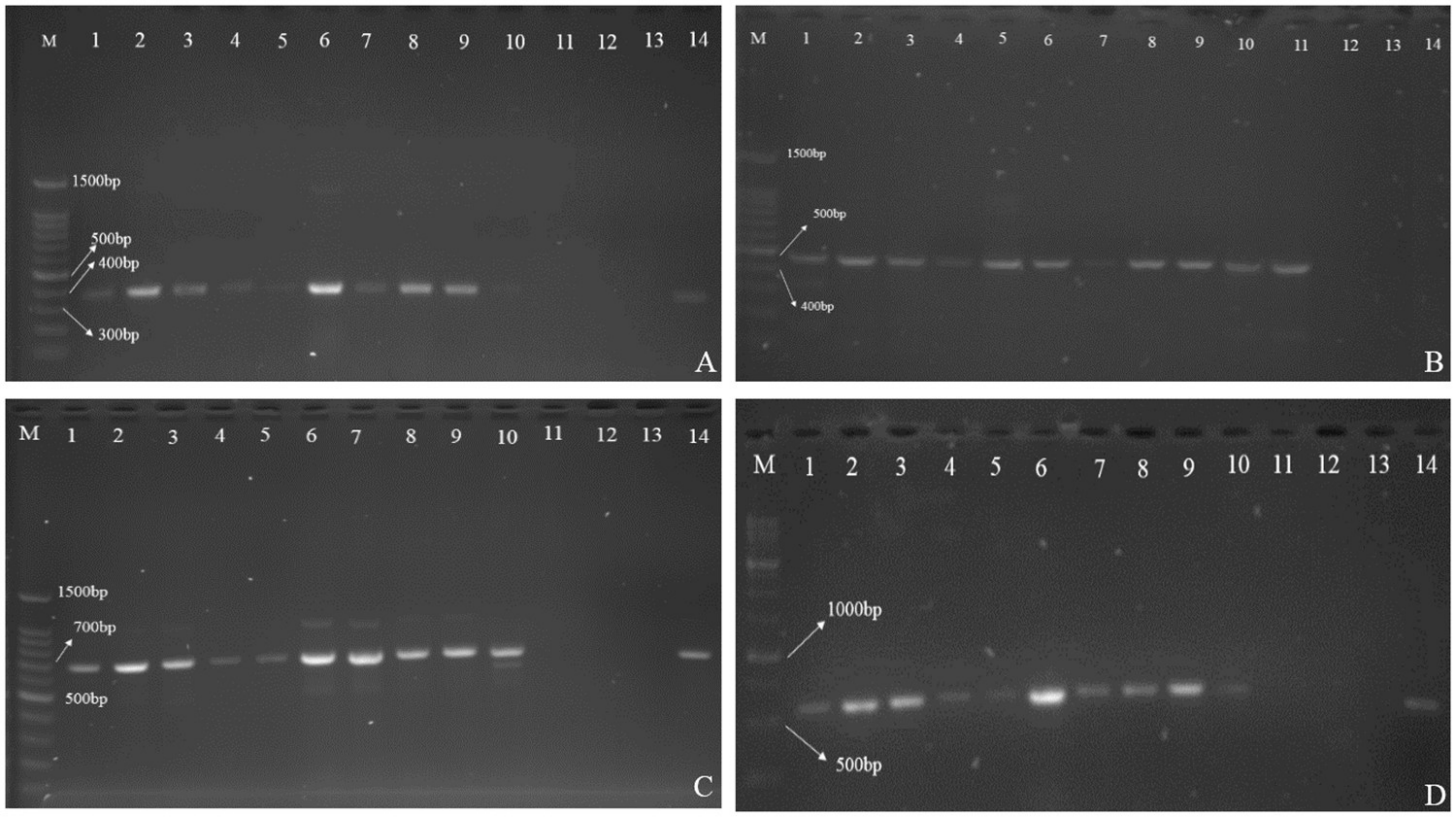
Gel electrophoresis of *lpx* genes. A: *lpxA* gene (356bp). M: DNA marker, 1–10: ECajums1-10, 11 and 12: ECajums 12 and 15, 13: negative control: *A*. *baumannii* type strain ATCC 19606, 14: Positive control: *E*. *coli* type strain ATCC 25922. B: *lpxB* gene (458bp). M: DNA marker, 1: Positive control: *E*. *coli* type strain ATCC 25922, 2–11: ECajums1-10, 12 and 13: ECajums 12 and 15, 14: negative control: *A*. *baumannii* type strain ATCC 19606. C: *lpxC* gene (689bp). M: DNA marker, 1–10: ECajums1-10, 11 and 12: ECajums 12 and 15, 13: negative control: *A*. *baumannii* type strain ATCC 19606, 14: Positive control: *E*. *coli* ATCC 25922. D: *lpxD* gene (574bp). M: DNA marker, 1–10: ECajums1-10, 11 and 12: ECajums 12 and 15, 13: negative control: *A*. *baumannii* type strain ATCC 19606, 14: Positive control: *E*. *coli* ATCC 25922.

Intriguingly, none of the 18 investigated strains presented rLPS or deletion in the according *lpx* genes (*lpxA*, *lpxB*, *lpxC* and *lpxD*) (Fig 2).

**Fig 3:**
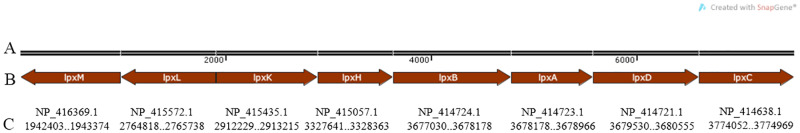
Analysis of the *lpx* coding cluster genes using data from *E*. *coli* K-12 substr. MG1655 (NCBI Reference Sequence: NZ_CP032667.1). A: The molecular size of the *lpx* coding cluster genes, B: The *lpx* coding cluster genes, their arrangement and directions, C: The lpx coding cluster genes NCBI code and their exact positions in the genome.

The lipid A coding cluster genes are concentrated in two distinct region of the genome (Fig 3), separated by 349kb. The second region was evaluated in this study.

**Fig 4:**
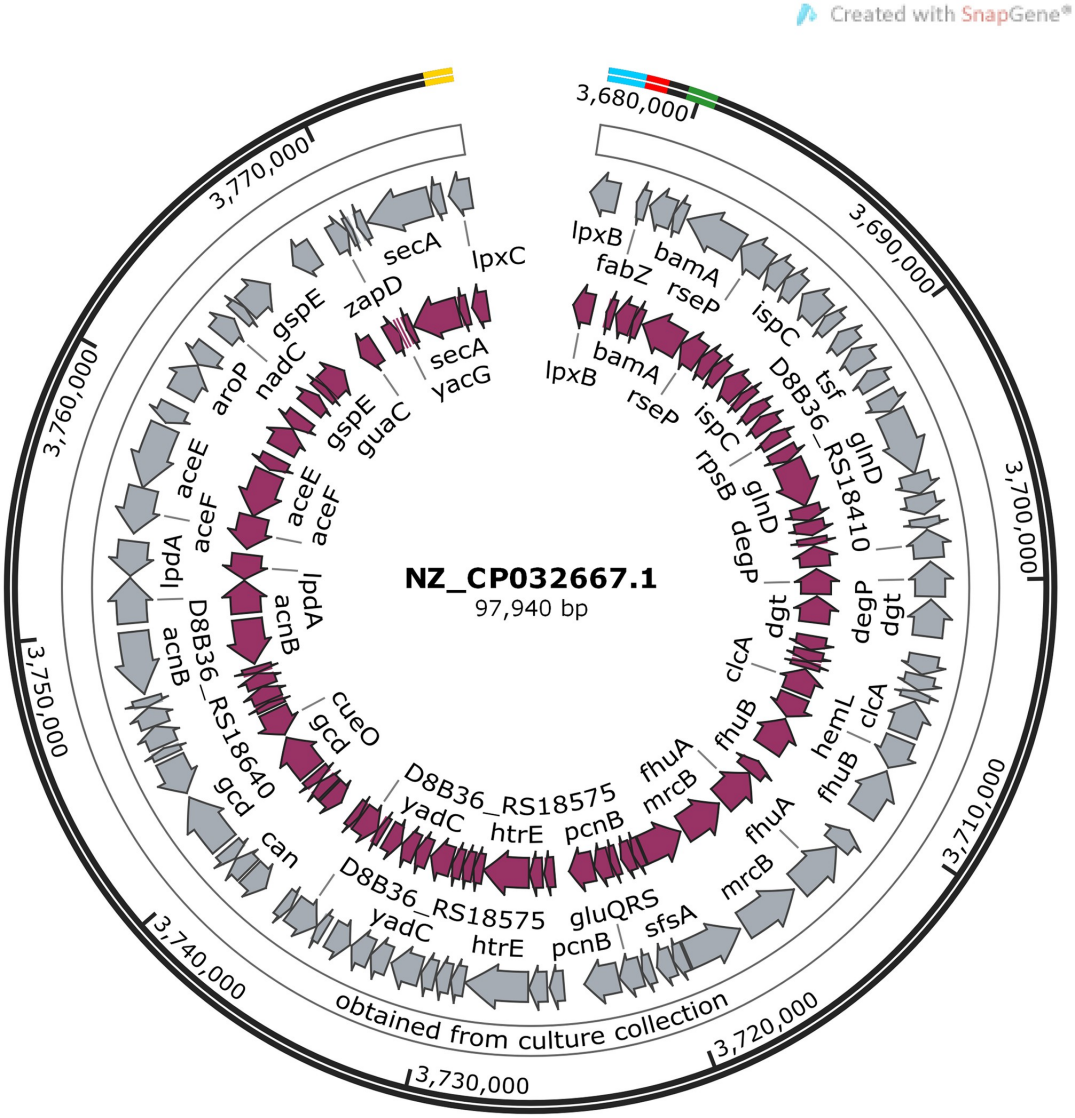
Analysis of the *lpxA*, *lpxB*, *lpxC*, *and lpxD* genes and the associated adjacent genes using data from *E*. *coli* K-12 substr. MG1655 (NCBI Reference Sequence: NZ_CP032667.1). The purple circle is showing the data from NZ_CP032667.1, and the gray circle is presenting data obtained from culture collection. The outer black circle is showing the exact positions of the *lpxA*, *lpxB*, *lpxC*, and *lpxD* genes among NZ_CP032667.1 genome (Yellow: *lpxC*, Blue: *lpxB*, Red: *lpxA*, Green: *lpxD*).

The genes *lpxB*, *lpxA*, and *lpxD* are near each other while, *lpxC* is in a significant distance from the other three family members and separated by 71 genes including 93kb (Fig 4).

By using the online Operon mapper (http://biocomputo.ibt.unam.mx/operon_mapper/) [27], it was determined that the genes *lpxB*, *lpxA*, and *lpxD* are functioning as an operon along with other 11 adjacent genes including *fabZ* which separate the *lpxB* and *lpxA* genes. By using the Tandem Repeats Finder online tool [28], we detected two regions for variable number tandem repeats (VNTRs) among these adjacent genes. The first was located between the positions 74637–74857 which it had a period size of 96 bp and copy number of 2.3. The second tandem repeat region was detected in the positions 78039–78781 which its period size was 309 bp and it had a copy number of 2.4.

By analyzing the *lpx* genes (*lpxA*, *lpxB*, *lpxC*, and *lpxD*) sequences using the MSA, we found multiple single nucleotide polymorphisms (SNPs). In *lpxA*, 7 SNPs were observed which led to only one amino acid substitution (I119L). In *lpxB*, only 1 SNP was detected, while no amino acid substitution was observed. In *lpxC*, 9 SNPs were detected but no amino acid substitution was occurred. In *lpxD*, we observed 28 SNPs, which led to only one amino acid substitution (I224V). Partial sequences of *lpx* genes from four colistin resistant *E*. *coli* isolates (ECajums1, ECajums9, ECajums26 and ECajums30) were deposited to the GenBank under the subsequent accession numbers: *lpxA* (MT196001-MT196004), *lpxB* (MT195997-MT196000), *lpxC* (MT195993-MT195996), and *lpxD* (MT195989-MT195992).

In regards to the colistin-resistant strains, we found that six out of the 20 resistant isolates (30%) were harbouring *mcr-1* but not *mcr-2*. Two of these six isolates, ECajums16 and ECajums25 with an MIC for colistin of ˃256 μg/ml and 2 μg/ml, respectively, had lost their LPS biosynthesis cluster (*lpxA*, *lpxB*, *lpxC* and *lpxD*) (Table 2). However, they harbored the *mcr-1* gene, but it appeared that colistin resistance was due to the loss of the cellular target in these isolates. The other four isolates, ECajums9, ECajums10, ECajums21, and ECajums26 had an MIC of 2.5, 4, 32, and ˃256 μg/ml, respectively, and showed intact sLPS (Table 2). Further investigation of the *lpx* genes revealed that these four isolates had no mutation in the *lpx* biosynthetic cluster (*lpxA*, *lpxB*, *lpxC* and *lpxD*). Next, we examined the role of *mcr-1* in resistance against colistin in these isolates. Heterologous expression of *mcr-1* in *E*. *coli* DH5α increased the MIC from 0.25 μg/ml to 2.5 μg/ml, conferring the role of *mcr-1* in resistance to colistin among these four isolates. Intriguingly, from the 14 colistin-resistant isolates not harbouring *mcr* genes, six presented no LPS and were disrupted in the *lpx* biosynthetic cluster. The colistin MICs ranged from 2 μg/ml to ˃256μg/ml. Three isolates had deletions in *lpx* genes, but not all of them showed the rLPS. The other five isolates showed sLPS types and no *mcr* gene (Table 2). Overall, we found five isolates with rLPS and six isolates were negative for LPS production. Both phenotypes were statistically significant related to the colistin resistance (P-value ≤ 0.05).

The occurrence of colistin-resistant strains has severe consequences for its clinical suitability [29]. There are increasing reports of colistin resistance among Gram-negative bacteria [30]. A study conducted by Rossia *et al*. in Brazil showed that within five years, 4% (n = 1346) of Gram-negative bacteria became colistin-resistant. Among these, *Enterobacteriaceae* were the most frequent (86.1%) pathogens [31]. In Iran, several reports presented colistin resistance among *Enterobacteriaceae*; an alarming situation for the health care system [24, 32]. In Europe, colistin resistance rates appear to be much lower than in developing countries. A study in Italy showed that among 3,902 clinical isolates of *E*. *coli*, only 0.5% were resistant to colistin [33]. There is no report on the resistance rate of Gram negative bacteria against Colistin from USA.

Resistance to colistin can occur via chromosomal mutations or the uptake of exogenous DNA, such as plasmids carrying colistin resistance genes (*mcr*). Indeed, there are emerging reports of plasmid-borne *mcr* genes, which provide a mechanism for rapid dissemination [34]. The first occurrence of the *mcr-1* gene was reported in China in 2015 [35]; the first report in the United States in 2016 [36]. In Iran, Moosavian and Emam reported that among 64 colistin-resistant *Enterobacteriaceae*, 1.7% (n = 8) of the isolates were already harbouring the *mcr-1* gene [24]. These reports indicating a global spread, and a new massive challenge for the health systems around the world. The *mcr-2* gene is a rare variant of *mcr-1* and is found only in Belgium. The less-related *mcr-3*, *mcr-4*, and *mcr-5* were identified in *E*. *coli* and *Salmonella* [20–22].

Interestingly, there have been other reports showing colistin-resistant isolates not harbouring *mcr* genes. In the study by Manohar *et al*., 24 colistin-resistant *Klebsiella pneumoniae* isolates were investigated and none of them carried *mcr-1* or *mcr-2* [37]. Potentially, this could be due to the loss of LPS as recently shown by mutations in lipid A biosynthesis genes in *E*. *coli* [38]. This strongly indicates that the interaction of colistin with LPS is critical for the bactericidal action of colistin against *E*. *coli*.

Colistin resistance due to the loss of LPS increases the susceptibility to other antibiotics. This could be applied in combined antibiotic-therapy regimens containing colistin and a second antibiotic effective against colistin-resistant, LPS-deficient Gram-negative bacteria and such a method may develop the employment of colistin as a good antimicrobial against MDR *Enterobacteriaceae* [39].

In summary, LPS derived from clinical *E*. *coli* isolates showed the rough type (sLPS) and was associated with a colistin-resistant phenotype. However, other resistant isolates had deletions in *lpx* genes and showed no LPS production. This is in accordance with other reports, where insertions such as *ISAba11* were shown to be associated with colistin resistance and loss of the LPS in other Gram-negative bacteria [40]. The detection of SNPs and VNTRs in *lpx* and adjacent genes confirmed the hyper-mutability within these regions [41].

## Conclusions

The complete loss of the LPS and/or production of the rLPS could lead to colistin resistance among *E*. *coli* clinical isolates not harbouring the *mcr-1* gene. It is due to the loss of the "lipid A", which is the molecular target for this antibiotic. Another reason could be the reduced affinity of the drug to its molecular target as a results of decreased net negative charge because of deletion of AgO chains in the rLPS.

## Supporting information

S1 Table Demographic data of 38 *Escherichia coli* clinical isolates used in this study. (DOCX)

S2 Table Distribution of absolute and relative frequency of 351 *Escherichia coli* clinical isolates from Ahvaz teaching hospitals used in this study. (DOCX)

S3 Table Absolute and relative abundance of 351 *Escherichia coli* clinical isolates used in this study based on isolation from different clinical specimens. (DOCX)

S1 File *lpxA* genes annotation file. (TXT)

S2 File *lpxB* genes annotation file. (TXT)

S3 File *lpxC* genes annotation file. (TXT)

S4 File *lpxD* genes annotation file. (TXT)

S1 Data (PDF)

S1 Fig Distribution of absolute and relative abundance of 351 *Escherichia coli* clinical isolates used in this study based on gender pf patients. (DOCX)

S2 Fig Distribution of relative frequency of 351 *Escherichia coli* isolates used in this study based on isolation from different wards of hospitals. (DOCX)

S3 Fig Antibiotic resistance pattern of 351 *Escherichia coli* isolates used in this study. (DOCX)

S4 Fig (TIF)

S5 Fig (TIF)

S6 Fig (TIF)

S7 Fig (TIF)

S8 Fig (TIF)

S9 Fig (TIF)

S10 Fig (TIF)

S11 Fig (TIF)

S12 Fig (TIF)

S13 Fig (TIF)

S14 Fig (TIF)
