# Supplementary material for: Rough-type and loss of the LPS due to lpx genes deletions are associated with colistin resistance in multidrug-resistant clinical Escherichia coli isolates not harbouring mcr genes
Source: PLoS One. 2020 May 20;15(5):e0233518. doi: 10.1371/journal.pone.0233518 (PMC7239443; doi:10.1371/journal.pone.0233518)
Supplement: S1 Table — (DOCX) [file pone.0233518.s001.docx]

| **Isolates** | **Gender of the patient** | **Hospital** | **Clinical Specimen** | **Ward** | **Drug Resistance Pattern** |
| --- | --- | --- | --- | --- | --- |
| ECajums1 | F | Golestan | Urine | OPC | T, CIP,CAZ |
| ECajums2 | F | Golestan | Urine | OPC | AT |
| ECajums3 | M | Razi | Urine | ICU | CAZ |
| ECajums4 | F | Abozar | Urine | ED | - |
| ECajums5 | F | Golestan | Urine | URO | AK, CIP, T, CAZ, AT |
| ECajums6 | F | Golestan | Urine | URO | AK, CIP, T, CAZ, AT |
| ECajums7 | M | Abozar | Urine | ICU | T, AT, CAZ |
| ECajums8 | F | Abozar | Urine | OPC | CAZ |
| ECajums9 | M | Abozar | Urine | NICU | T, AT, CAZ |
| ECajums10 | F | Golestan | Urine | ICU | T, CAZ |
| ECajums11 | F | Razi | Urine | ICU | AK, CIP, T, CAZ, IMI, AT |
| ECajums12 | F | Golestan | Urine | ICU | AK, CIP, T, CAZ, AT |
| ECajums13 | F | Razi | Urine | OPC | AT, CIP, CAZ |
| ECajums14 | F | Abozar | Urine | OPC | T, AK, CIP, CAZ |
| ECajums15 | F | Golestan | Urine | ICU | AT, T, CAZ, IMI |
| ECajums16 | F | Golestan | Urine | ICU | AT, T, CAZ, IMI |
| ECajums17 | F | Abozar | Urine | URO | CAZ, IMI |
| ECajums18 | M | Golestan | Blood | NEU | T, AT, CAZ, IMI |
| ECajums19 | F | Abozar | Urine | URO | T, AT, CAZ, IMI |
| ECajums20 | M | Golestan | Urine | URO | T, CIP, IMI, CAZ |
| ECajums21 | M | Golestan | Urine | OPC | CAZ, IMI |
| ECajums22 | F |  | Urine | OPC | AK, T, CAZ, IMI |
| ECajums23 | F | Razi | Urine | ID | CAZ, IMI |
| ECajums24 | M | Razi | Urine | ID | T, AT, CIP, CAZ, IMI |
| ECajums25 | M | Abozar | Urine | NICU | T, CIP, CAZ, IMI |
| ECajums26 | M | Golestan | Urine | ICU | AT, T, CAZ, IMI, CIP |
| ECajums27 | F | Razi | Urine | ID | CAZ |
| ECajums28 | M | Golestan | Discharge | URO | T, CIP, CAZ |
| ECajums29 | F | Golestan | Urine | OPC | T, CIP, CAZ |
| ECajums30 | F | Razi | Urine | ID | AT, IMI, CIP |
| ECajums31 | M | Abozar | Urine | ICU | T, CIP, IMI, CAZ |
| ECajums32 | F | Abozar | Urine | OPC | T, CAZ |
| ECajums33 | F | Abozar | Urine | OPC | AT, T, CAZ |
| ECajums34 | M | Golestan | Urine | OPC | AT, CIP |
| ECajums35 | M | Abozar | Urine | ICU | AT |
| ECajums36 | F | Golestan | Urine | ICU | T, CIP, CAZ |
| ECajums37 | M | Razi | Urine | ICU | AK, CIP, T, CAZ, IMI, AT |
| ECajums38 | F | Golestan | Urine | URO | AT, CIP |

Demographic data of 38 *Escherichia coli* clinical isolates used in this study

Abbreviations: F: female, M: male, ICU: intensive care unit, URO: urology, ID: infectious disease, NICU: neonatal intensive care unit, OPC: out patients clinic, ED: emergency Department, NEU: neurology, CAZ: ceftazidime, T: tetracycline, AT: azithromycin, CIP: ciprofloxacin, IMI: imipenem, AK: amikacin.
