# Supplementary material for: Rough-type and loss of the LPS due to lpx genes deletions are associated with colistin resistance in multidrug-resistant clinical Escherichia coli isolates not harbouring mcr genes
Source: PLoS One. 2020 May 20;15(5):e0233518. doi: 10.1371/journal.pone.0233518 (PMC7239443; doi:10.1371/journal.pone.0233518)
Supplement: S2 Table — (DOCX) [file pone.0233518.s002.docx]

| *E. coli* | Hospital |
| --- | --- |
| No % |  |
| 59 207 | Golestan |
| 35 123 | Abozar |
| 6 21 | Razi |
| 100 351 | Total |

Distribution of absolute and relative frequency of 351 *Escherichia coli* clinical isolates from Ahvaz teaching hospitals used in this study.
