# Supplementary material for: Rough-type and loss of the LPS due to lpx genes deletions are associated with colistin resistance in multidrug-resistant clinical Escherichia coli isolates not harbouring mcr genes
Source: PLoS One. 2020 May 20;15(5):e0233518. doi: 10.1371/journal.pone.0233518 (PMC7239443; doi:10.1371/journal.pone.0233518)
Supplement: S3 Table — (DOCX) [file pone.0233518.s003.docx]

| % | No. of isolates | Samples |
| --- | --- | --- |
| 84.6  5.7  4.3  2.9  1.7  0.8 | 297  20  15  10  6  3 | Urine  Trachea  Blood  Wound  Discharge  CSF |
| 100 | 351 | Total |

Absolute and relative abundance of 351 *Escherichia coli* clinical isolates used in this study based on isolation from different clinical specimens
