# Supplementary material for: Rough-type and loss of the LPS due to lpx genes deletions are associated with colistin resistance in multidrug-resistant clinical Escherichia coli isolates not harbouring mcr genes
Source: PLoS One. 2020 May 20;15(5):e0233518. doi: 10.1371/journal.pone.0233518 (PMC7239443; doi:10.1371/journal.pone.0233518)
Supplement: S1 Data — (PDF) [file pone.0233518.s008.pdf]

ENGLISH EDIT WWW.NativeEnglishEdit.com

EDITORIAL CERTIFICATE LETTER

This document is to certify that the manuscript listed below was edited for proper English language, grammar, punctuation, spelling, and overall style by one of the highly qualified subject-expert native English speaking editors at **NativeEnglishEdit.com**

The substantive content of the article mentioned below remains the full responsibility of the author/authors:

Rough-type and loss of the LPS due to lpx genes deletions are associaed with colistin resistance among Multiple Drug Resistant Escherichia coli clinical isolates not harbring mcr genes.

Mojtaba Moosavian, Nasrin Emam,Daniel Pletzer, Mohammad Savari

Native English Edit
www.NativeEnglishEdit.com

Documents receiving this certification should be English-ready for publication; however, the author has the ability to accept or reject our suggestions and changes.

This certificate may be verified at:

Native English Edit

www.birminghamresearchpark.co.uk/tenants/native-english-edit

www.NativeEnglishEdit.com

Birmingham Research Park
Edgbaston
Birmingham B15 2SQ
United Kingdom
