# Supplementary material for: Rough-type and loss of the LPS due to lpx genes deletions are associated with colistin resistance in multidrug-resistant clinical Escherichia coli isolates not harbouring mcr genes
Source: PLoS One. 2020 May 20;15(5):e0233518. doi: 10.1371/journal.pone.0233518 (PMC7239443; doi:10.1371/journal.pone.0233518)
Supplement: S1 Fig — (DOCX) [file pone.0233518.s009.docx]

Distribution of absolute and relative abundance of 351 *Escherichia coli* clinical isolates used in this study based on gender pf patients
