# Supplementary material for: Rough-type and loss of the LPS due to lpx genes deletions are associated with colistin resistance in multidrug-resistant clinical Escherichia coli isolates not harbouring mcr genes
Source: PLoS One. 2020 May 20;15(5):e0233518. doi: 10.1371/journal.pone.0233518 (PMC7239443; doi:10.1371/journal.pone.0233518)
Supplement: S3 Fig — (DOCX) [file pone.0233518.s011.docx]

Antibiotic Resistance Pattern of 351 *Escherichia coli* Isolates used in this study

Abbreviations: CAZ: Ceftazidime, T: Tetracycline, AT: Azithromycin, CIP: Ciprofloxacin, IMI: Imipenem, AK: Amikacin, COL: Colistin.
