# Supplementary figures and images for: Rough-type and loss of the LPS due to lpx genes deletions are associated with colistin resistance in multidrug-resistant clinical Escherichia coli isolates not harbouring mcr genes
Source: PLoS One. 2020 May 20;15(5):e0233518. doi: 10.1371/journal.pone.0233518 (PMC7239443; doi:10.1371/journal.pone.0233518)

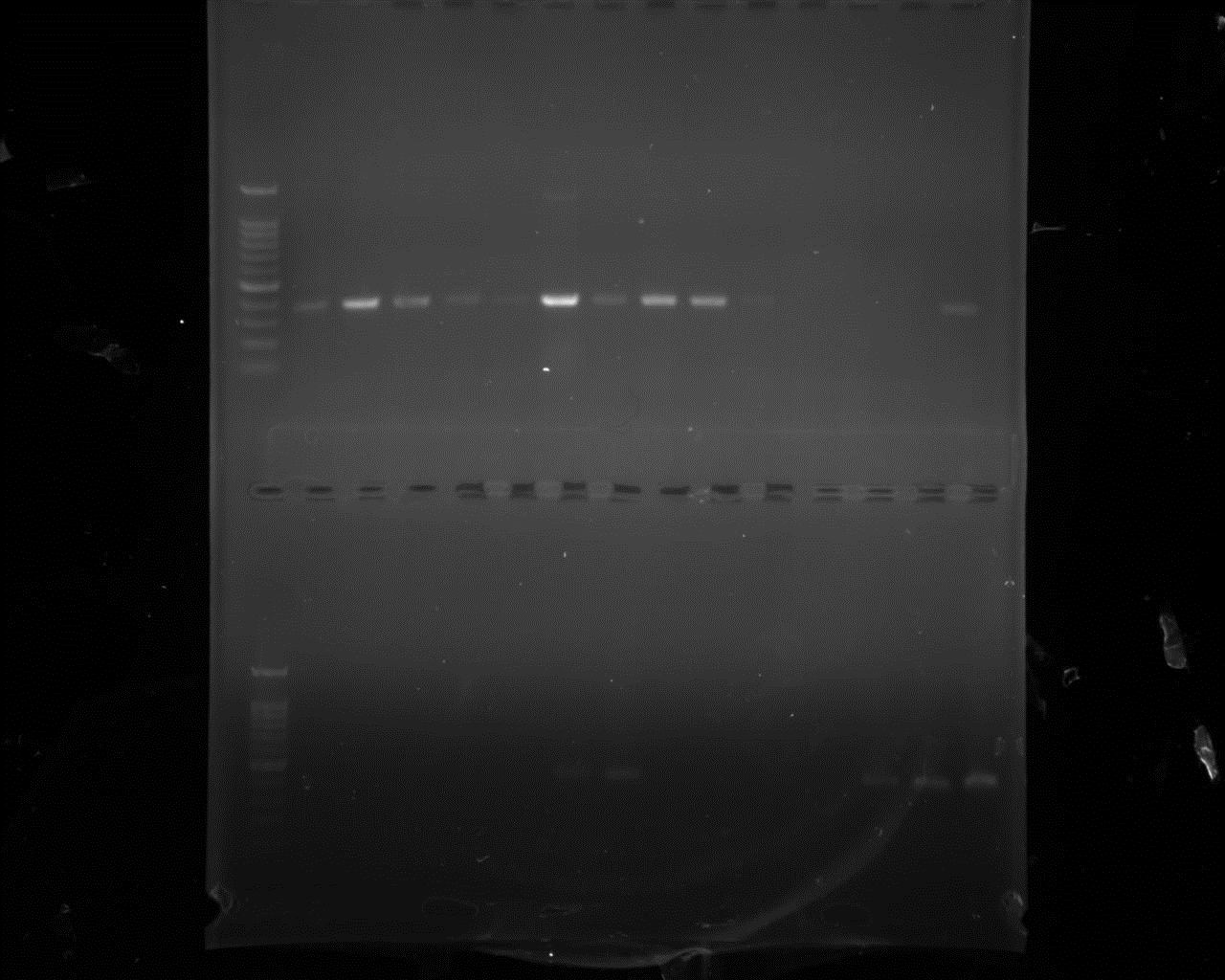

Supplement: S4 Fig — (TIF) [file pone.0233518.s012.tif]

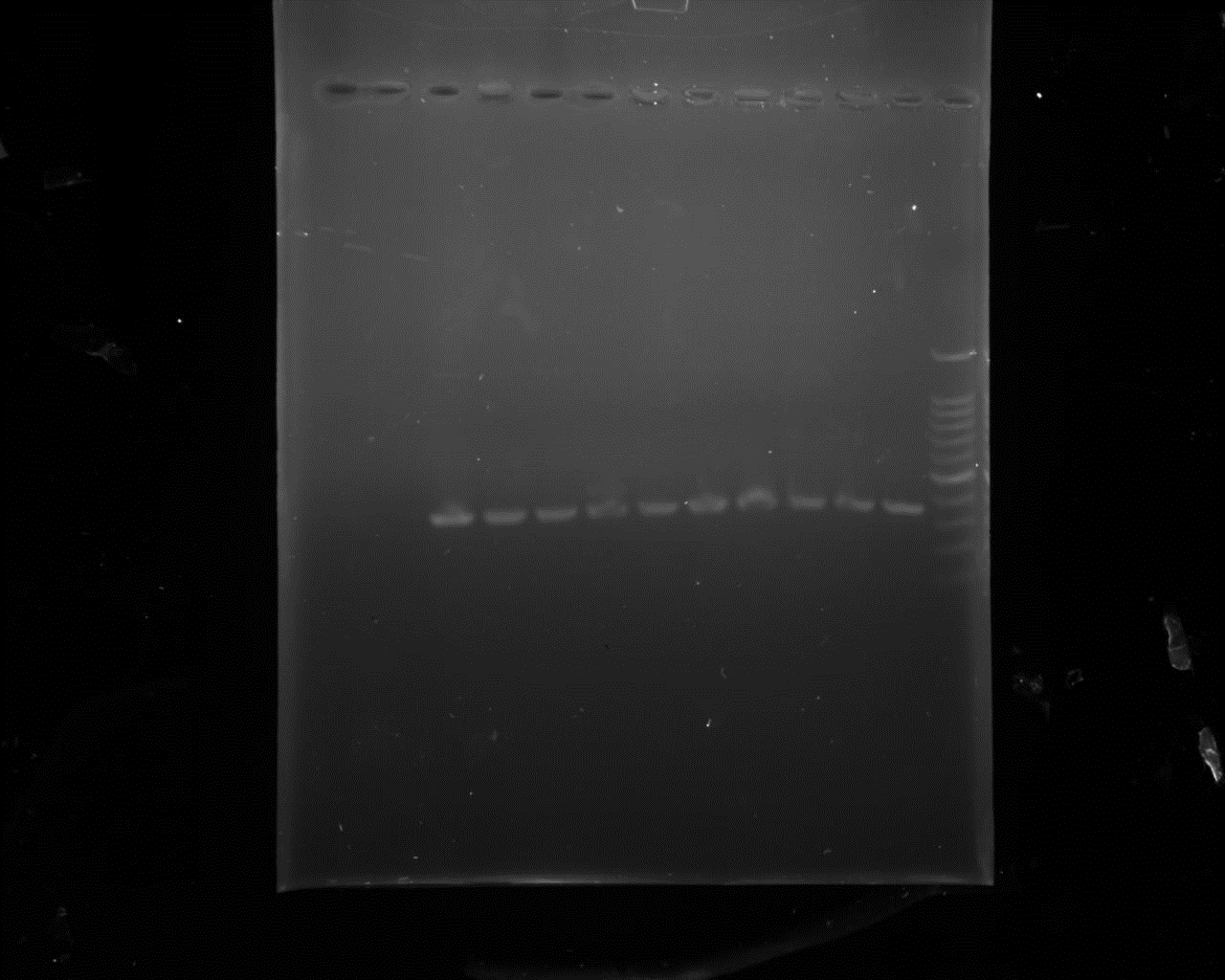

Supplement: S5 Fig — (TIF) [file pone.0233518.s013.tif]

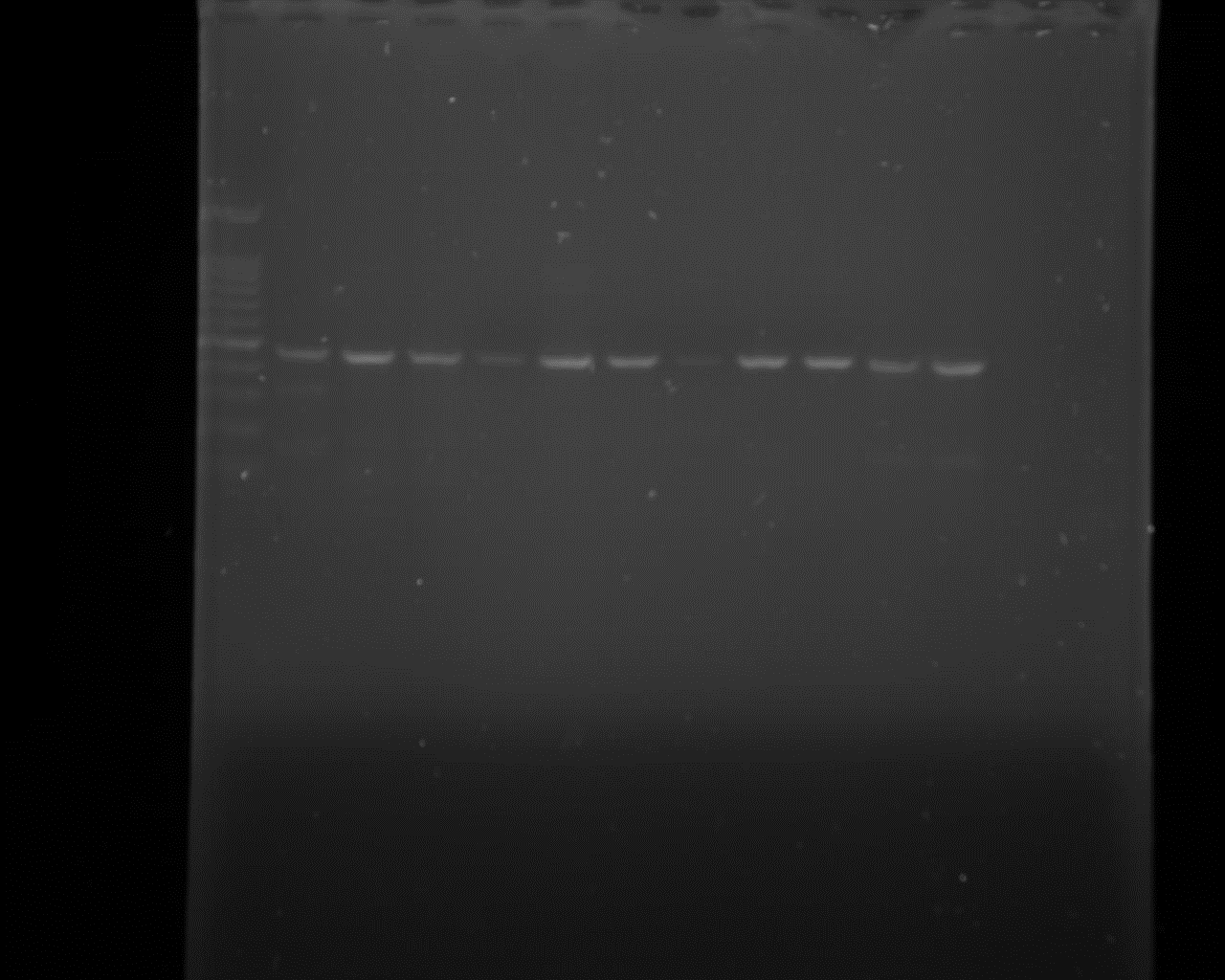

Supplement: S6 Fig — (TIF) [file pone.0233518.s014.tif]

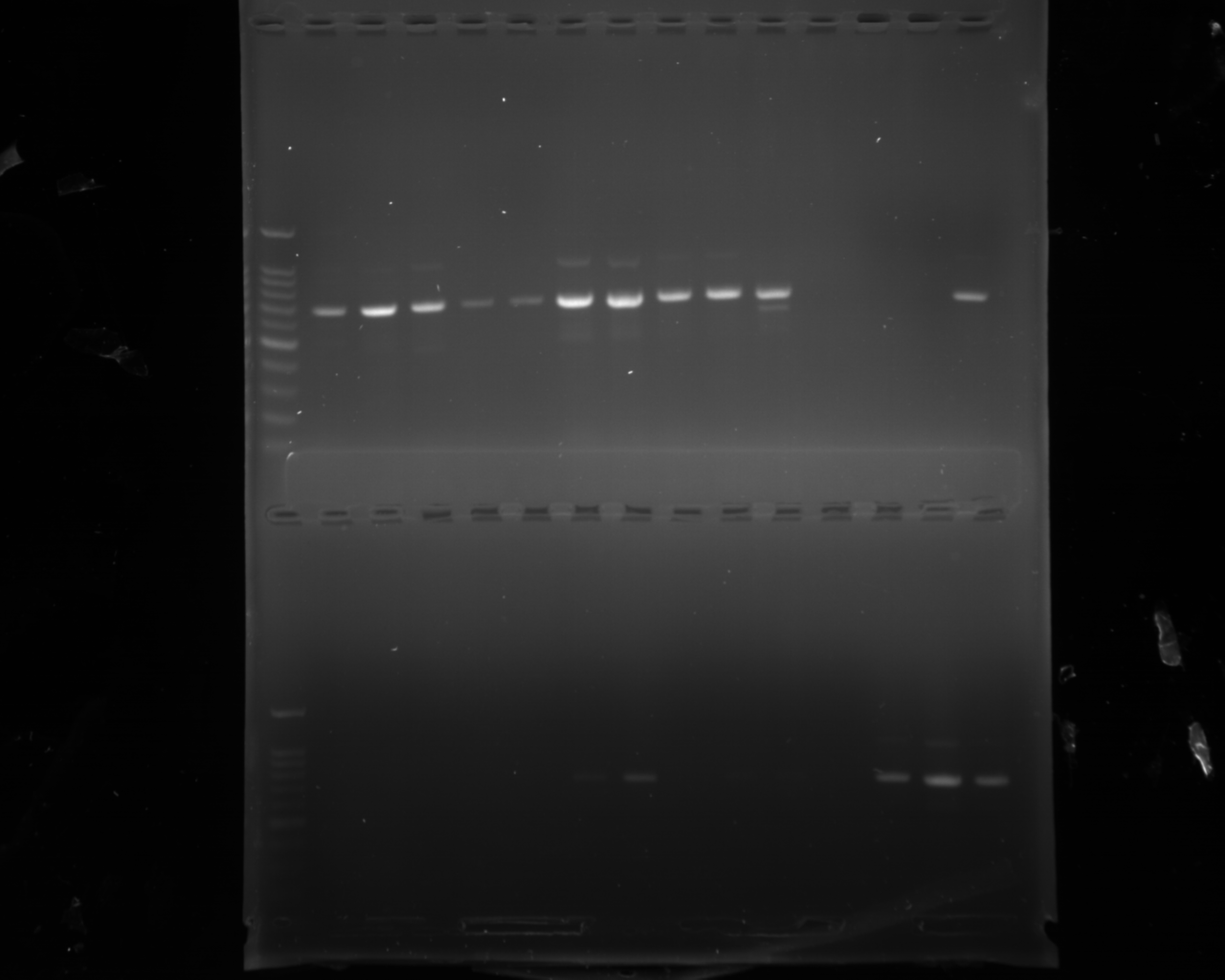

Supplement: S7 Fig — (TIF) [file pone.0233518.s015.tif]

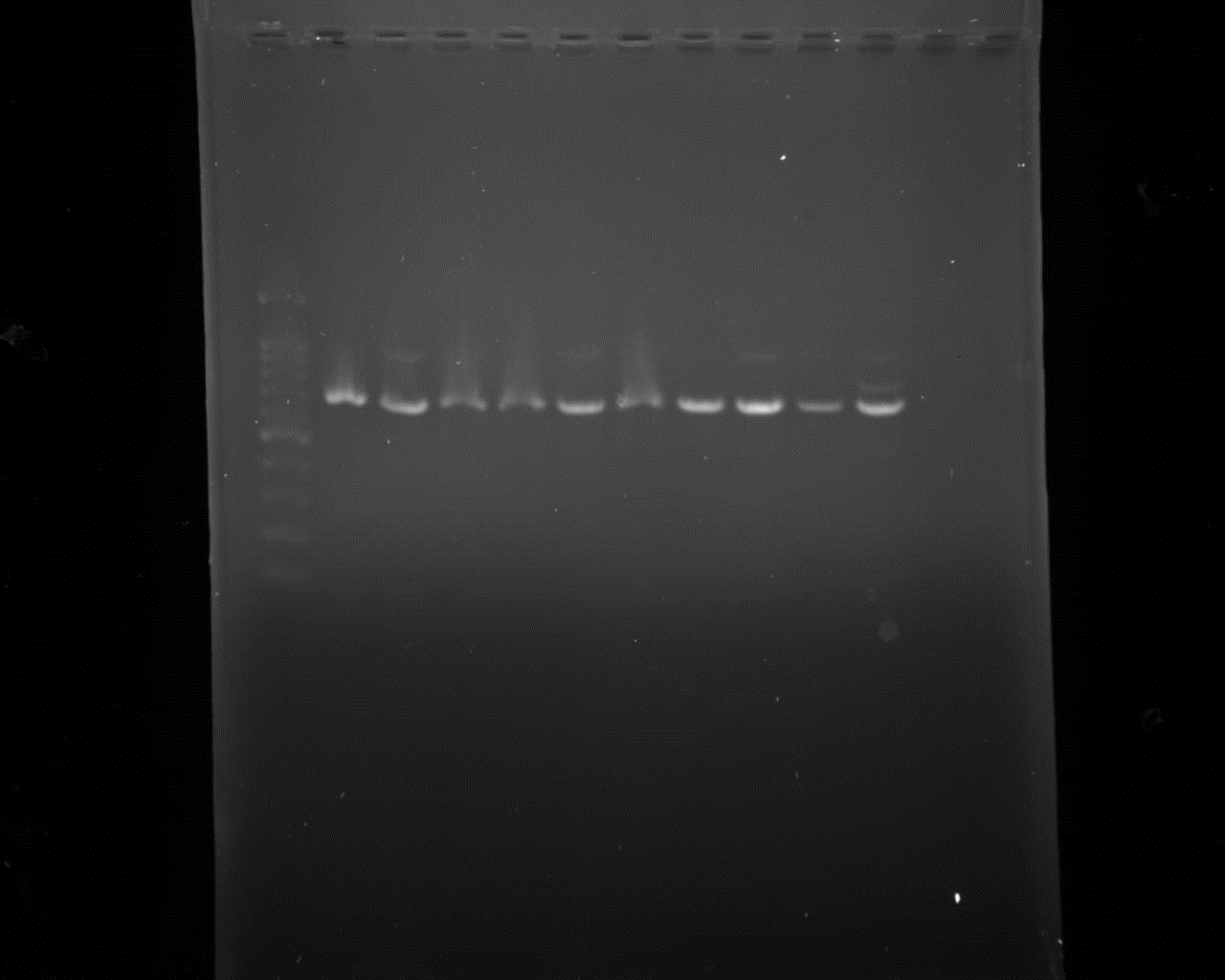

Supplement: S8 Fig — (TIF) [file pone.0233518.s016.tif]

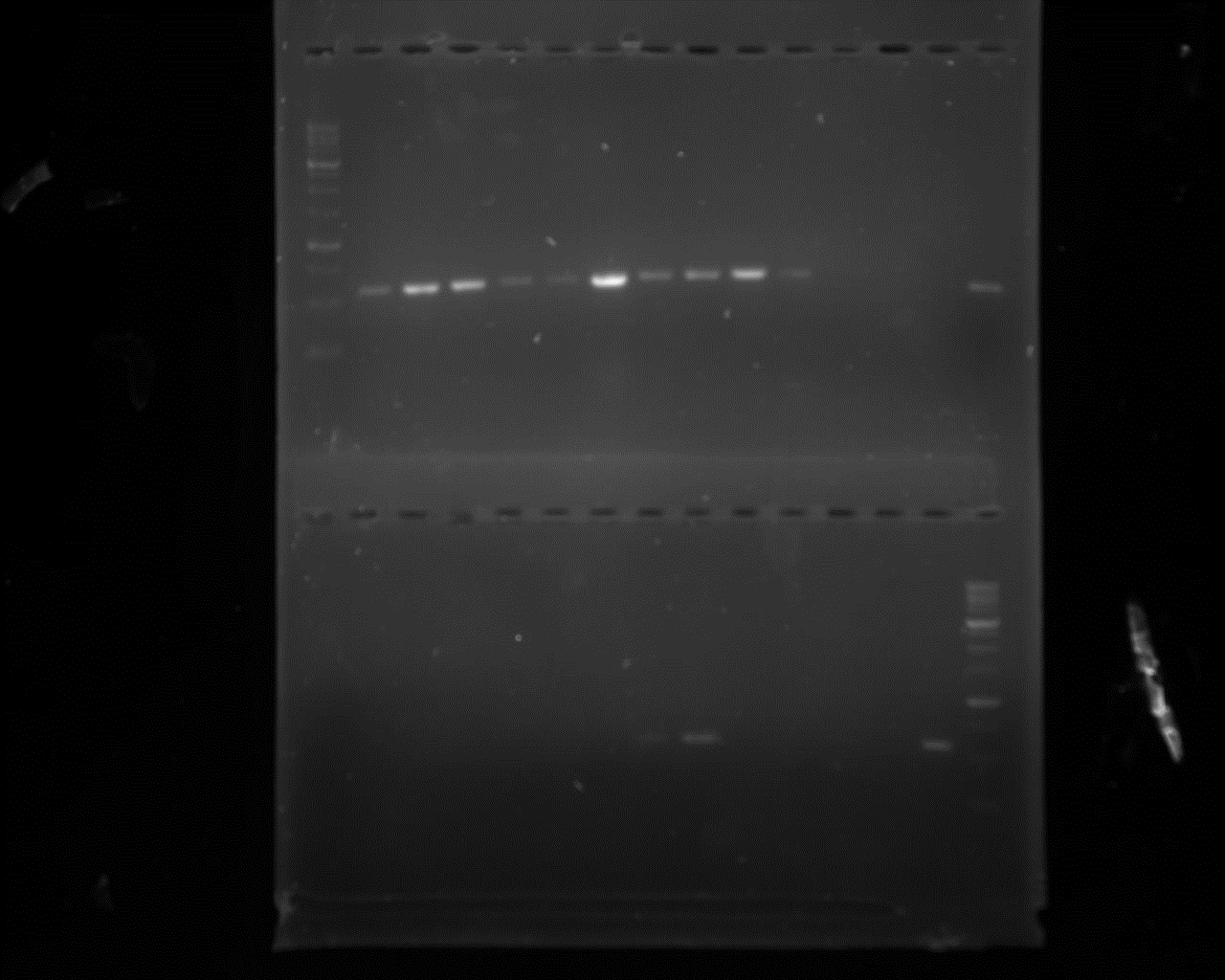

Supplement: S9 Fig — (TIF) [file pone.0233518.s017.tif]

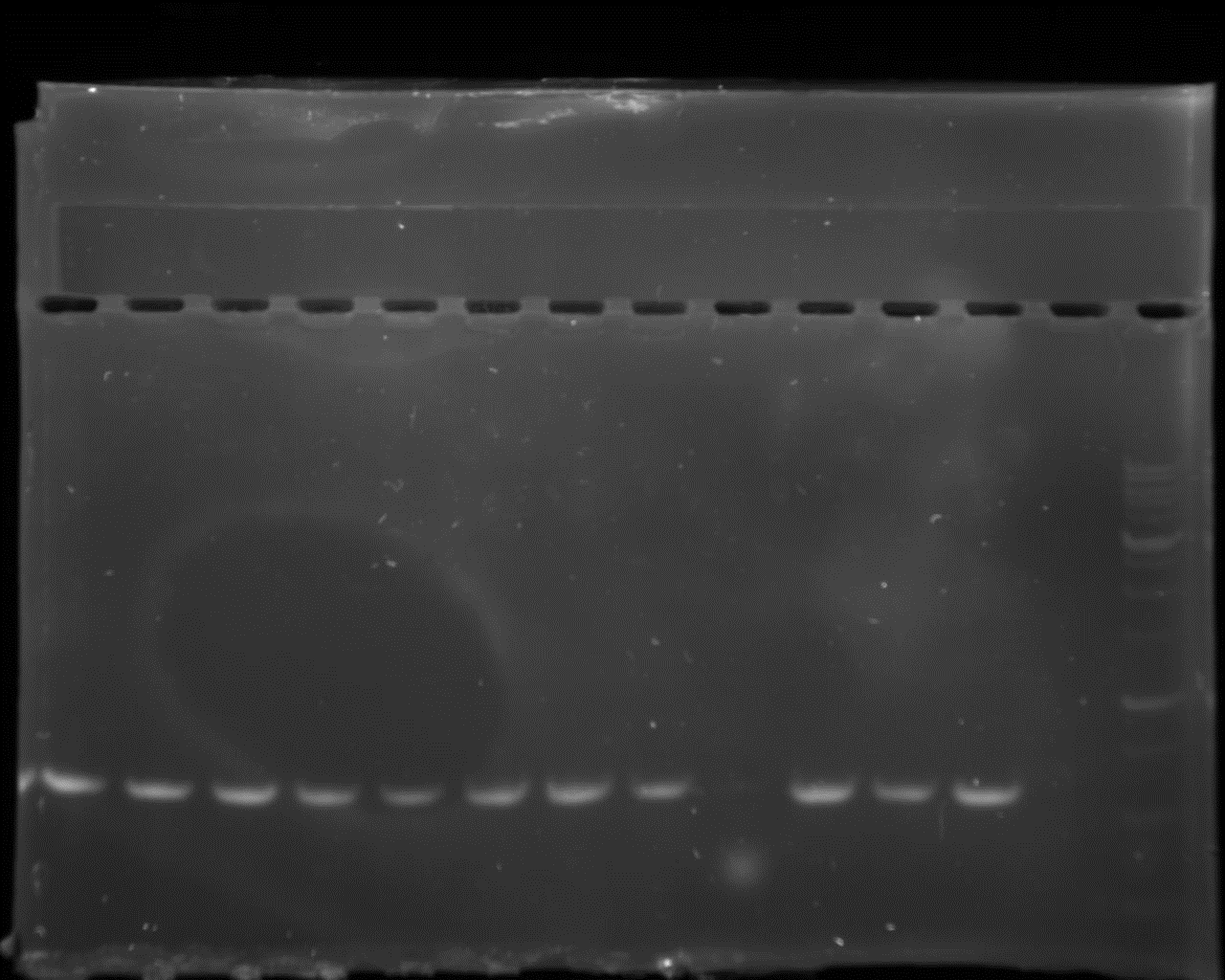

Supplement: S10 Fig — (TIF) [file pone.0233518.s018.tif]

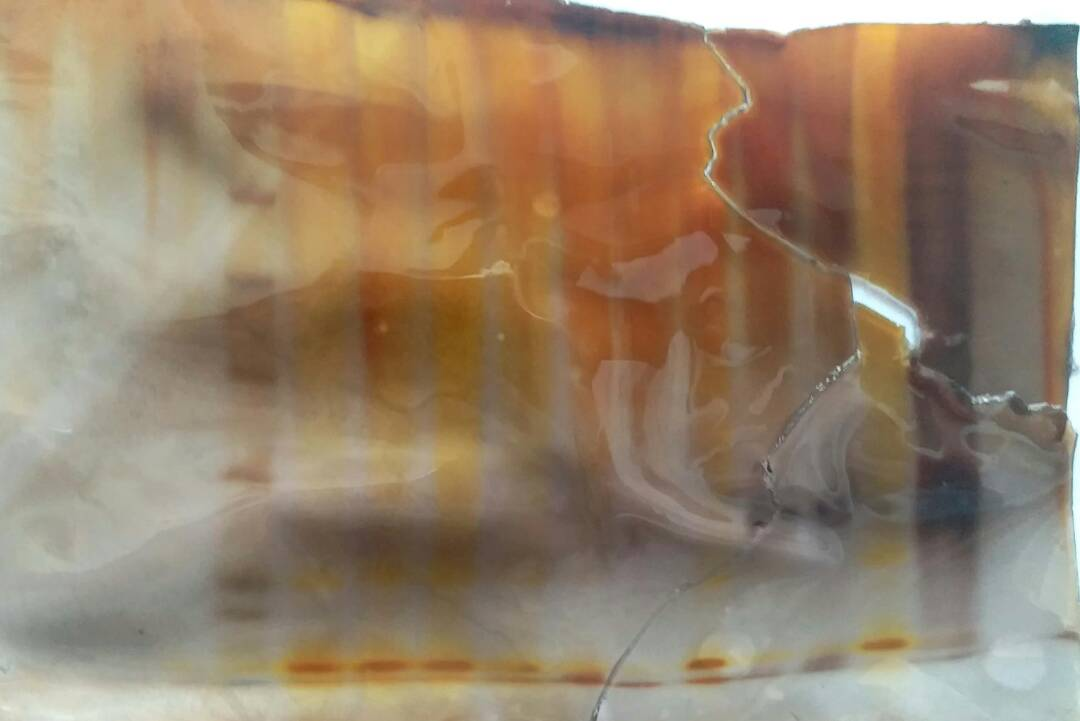

Supplement: S11 Fig — (TIF) [file pone.0233518.s019.tif]

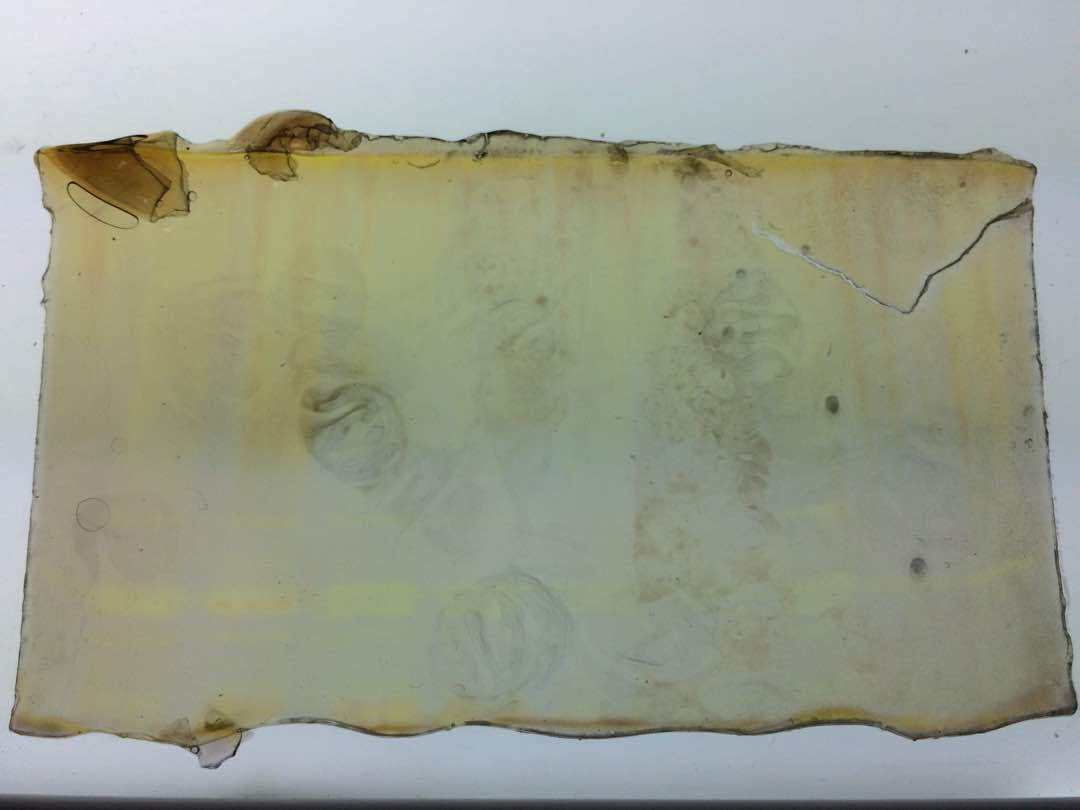

Supplement: S12 Fig — (TIF) [file pone.0233518.s020.tif]

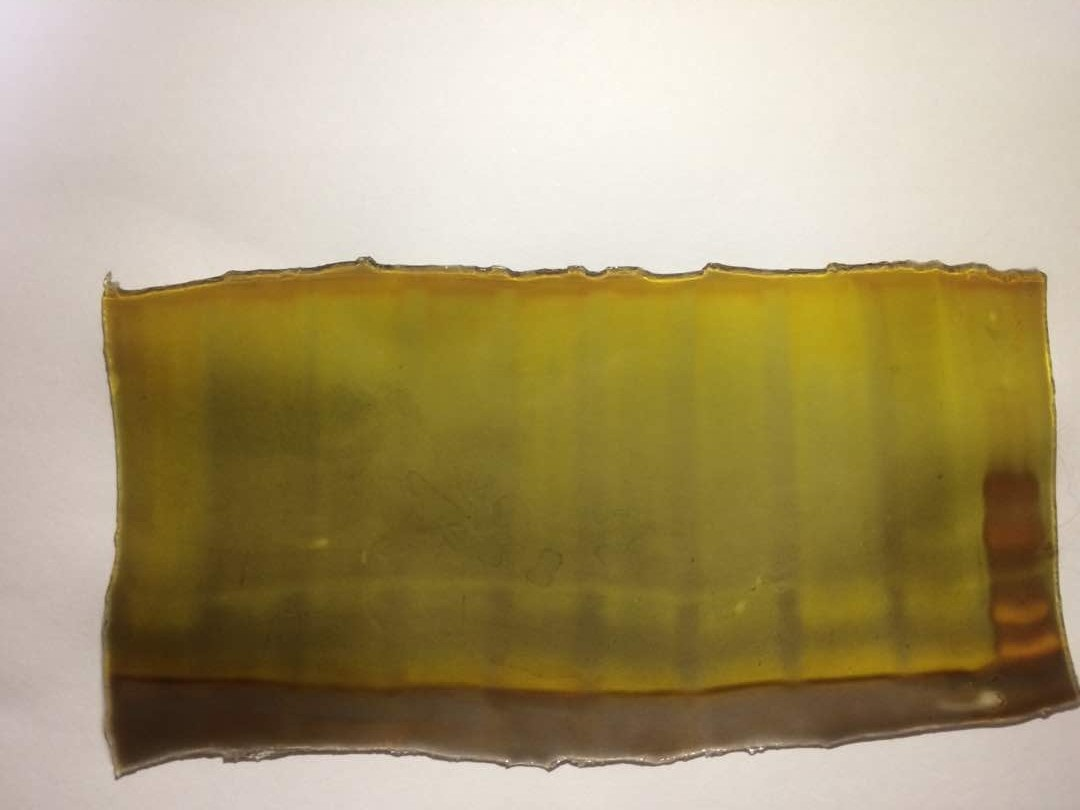

Supplement: S13 Fig — (TIF) [file pone.0233518.s021.tif]

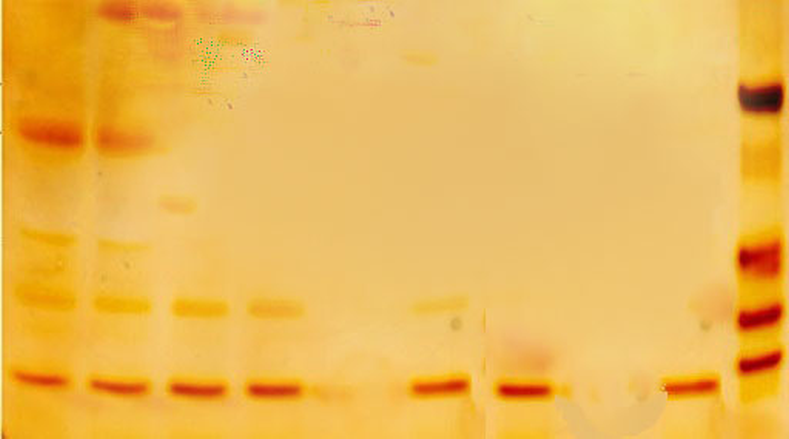

Supplement: S14 Fig — (TIF) [file pone.0233518.s022.tif]
